# DeepMIP-Eocene-p1: multi-model dataset and interactive web application for Eocene climate research

**DOI:** 10.1038/s41597-024-03773-4

**Published:** 2024-09-05

**Authors:** Sebastian Steinig, Ayako Abe-Ouchi, Agatha M. de Boer, Wing-Le Chan, Yannick Donnadieu, David K. Hutchinson, Gregor Knorr, Jean-Baptiste Ladant, Polina Morozova, Igor Niezgodzki, Christopher J. Poulsen, Evgeny M. Volodin, Zhongshi Zhang, Jiang Zhu, David Evans, Gordon N. Inglis, A. Nele Meckler, Daniel J. Lunt

**Affiliations:** 1 School of Geographical Sciences, University of Bristol, Bristol, UK; 2 Atmosphere and Ocean Research Institute, University of Tokyo, Kashiwa, Japan; 3 Department of Geological Sciences, Bolin Centre for Climate Research, Stockholm University, Stockholm, Sweden; 4 Aix Marseille Univ, CNRS, IRD, INRA, Coll France, CEREGE, Aix-en-Provence, France; 5 Climate Change Research Centre, University of New South Wales Sydney, Sydney, Australia; 6 Alfred Wegener Institute, Helmholtz Centre for Polar and Marine Research, Bremerhaven, Germany; 7 Laboratoire des Sciences du Climat et de l’Environnement, LSCE/IPSL, CEA-CNRS-UVSQ, Université Paris-Saclay, Gif-sur-Yvette, France; 8 Institute of Geography, Russian Academy of Sciences, Moscow, Russia; 9 ING PAN - Institute of Geological Sciences Polish Academy of Sciences, Research Center in Kraków, Biogeosystem Modelling Group, Kraków, Poland; 10 Department of Earth Sciences, University of Oregon, Eugene, Oregon USA; 11 Institute of Numerical Mathematics, Russian Academy of Sciences, Moscow, Russia; 12 NORCE Norwegian Research Centre, Bjerknes Centre for Climate Research, Bergen, Norway; 13 Department of Atmospheric Science, School of Environmental Studies, China University of Geosciences, Wuhan, China; 14 Climate & Global Dynamics Laboratory, NSF National Center for Atmospheric Research, Boulder, USA; 15 School of Ocean and Earth Science, University of Southampton, Southampton, UK; 16 Bjerknes Centre for Climate Research and Department of Earth Science, University of Bergen, Bergen, Norway

**Keywords:** Palaeoclimate, Palaeoceanography

## Abstract

Paleoclimate model simulations provide reference data to help interpret the geological record and offer a unique opportunity to evaluate the performance of current models under diverse boundary conditions. Here, we present a dataset of 35 climate model simulations of the warm early Eocene Climatic Optimum (EECO; ~ 50 million years ago) and corresponding preindustrial reference experiments. To streamline the use of the data, we apply standardised naming conventions and quality checks across eight modelling groups that have carried out coordinated simulations as part of the Deep-Time Model Intercomparison Project (DeepMIP). Gridded model fields can be downloaded from an online repository or accessed through a new web application that provides interactive data exploration. Local model data can be extracted in CSV format or visualised online for streamlined model-data comparisons. Additionally, processing and visualisation code templates may serve as a starting point for advanced analysis. The dataset and online platform aim to simplify accessing and handling complex data, prevent common processing issues, and facilitate the sharing of climate model data across disciplines.

## Background & Summary

Past climate changes provide an opportunity to better understand how key components of the climate system might change under anthropogenic greenhouse gas emissions and thus help constrain future climate change^1^. Comparisons with paleoclimate data allow us to evaluate climate models under atmospheric CO_2_ scenarios similar to those possible in the near future. Furthermore, these paleoclimate model simulations provide global, physically consistent reference data to support the interpretation of paleoclimatic data across a wide range of disciplines, e.g. in geology, biology, and geochemistry.

One of the most well-studied deep-time intervals with respect to model-data comparison is the early Eocene Climatic Optimum (EECO; ~53.3 to 49.1 million years ago^2^) as it provides an analogue for future very high emission scenarios^3^. It was characterised by atmospheric CO_2_ concentrations of ~1,500 ppmv^4^ and global mean surface temperatures (GMSTs) 10 to 16°C warmer than pre-industrial^5^. Several modelling studies have focused on improving our understanding of the mechanisms and implications of EECO warmth^6–10^ and ultimately motivated the formulation of the Eocene Modelling Intercomparison Project (EoMIP)^11^. While limited due to its opportunistic design, EoMIP nonetheless highlighted the possibility of using multi-model ensembles to systematically assess model-model and model-data differences in our understanding of Eocene climate.

Building on this potential, DeepMIP - the Deep-Time Model Intercomparison Project - was designed to provide a consistent framework to carry out coordinated EECO model experiments^12^. Eight modelling groups performed a total of 35 model simulations using the same paleogeographic and vegetation boundary conditions at a range of atmospheric CO_2_ concentrations (Table 1). These new simulations showed more consistent global mean surface temperatures across the ensemble and larger climate sensitivities compared to the EoMIP results^13^. The coordinated experiment set-up allowed a separation of the relative influence of changes in CO_2_ concentrations and non-CO_2_ boundary conditions (i.e. removal of land ice and prescribed vegetation) on the simulated surface temperatures. Non-CO_2_ boundary conditions alone lead to 3-5°C overall warming and contribute substantially to the reduced meridional temperature gradient, while higher CO_2_ levels drive global mean warming due to decreases in atmospheric emissivity. Importantly, three models (CESM1.2-CAM5, GFDL-CM2.1 and NorESM1-F) were able to produce absolute GMSTs and reduced meridional temperature gradients consistent with the geological record at CO_2_ concentrations within the reported range of EECO reconstructions (1170 to 2490 ppmv^14^).

**Table 1:** Summary of the available DeepMIP-Eocene model simulations in version 1.0 of the dataset. Experiment short names are defined in Table 2 and paleogeographies are shown in Fig. 1.

Model	Family	PI	×1	×1.5	×2	×3	×4	×6	×9	Geography	Reference
CESM1.2-CAM5	CESM	×	×			×		×	×	^29^	^13,32^
COSMOS-landveg-r2413	COSMOS	×	×			×	×			^29^	^13^
GFDL-CM2.1	GFDL	×	×		×	×	×	×		^29^	^13^
HadCM3B-M2.1aN	HadCM3	×	×		×	×				^29^	^13^
HadCM3BL-M2.1aN	HadCM3	×	×		×	×				^29^	^13^
INM-CM4-8	INMCM	×						×		^29^	^13^
IPSLCM5A2	IPSL	×		×		×				^29^	^13,40^
MIROC4m	MIROC	×	×		×	×				^29^	^13^
NorESM1-F	NorESM	×			×		×			^30^	^13^

The DeepMIP-Eocene ensemble has already been used in multiple studies, analysing specific aspects of the Eocene climate in more detail, e.g. the meridional temperature gradient^15^, the surface to deep ocean temperature relationship^16^, ocean circulation^17^, sea ice^18^, hydroclimate^19–23^, and the impact of mountains^24,25^. We anticipate continued interest in the DeepMIP-Eocene model data, both for model intercomparisons and for model-data syntheses, and aim to document the design of the dataset and streamline access to improve future reuse of the data. Although the use of large model ensembles is helpful in quantifying the influence of uncertainties in boundary conditions and limitations in model performance on the simulated Eocene climate, it also presents a technical hurdle in accessing and fully utilising the available data. The use of model-specific data standards, post-processing workflows and variable naming schemes can make the analysis and comparison of multi-model ensembles a tedious process or even lead to processing errors. The need for significant data processing expertise can therefore limit the benefits and wider use of these important data, particularly in non-modelling paleoclimatology disciplines.

Here, we build on the DeepMIP framework to address these issues and present standardised, quality-checked EECO model output to facilitate multi-model processing and analysis, both for model intercomparisons and model-data comparisons. We have reprocessed the output of a total of 26 EECO simulations at CO_2_ concentrations between ×1 and ×9 pre-industrial levels, together with their nine pre-industrial reference experiments, to generate a dataset of common climate variables with consistent temporal averaging, variable names and units across the ensemble. We follow the CMIP convention for variable names and units as closely as possible to take advantage of existing processing workflows, and use the ensemble spread to quantify the internal consistency of the output fields.

We provide two complementary ways of accessing the dataset, tailored to the most likely future use cases. First, the entire dataset is stored as global, gridded netCDF (network Common Data Form) files in the Centre for Environmental Data Analysis (CEDA) Archive and can be downloaded as individual files or in batch mode^26^. Combined with the consistent DeepMIP naming convention, this provides a more traditional, scriptable starting point for further analysis. This approach shares the goals of other existing infrastructure projects for sharing climate model data such as the Earth System Grid Federation (ESGF)^27^, but the limited scope and overall much smaller file sizes of this dataset allow us to use centralised, rather than distributed, data storage for greater user convenience. Second, we present an interactive web application to facilitate model-data comparisons of EECO surface temperatures and precipitation. This is a very common use case for paleoclimate model data, but also involves multiple processing steps and potential pitfalls, especially when working with a large model ensemble. Modern web technologies provide the opportunity for intuitive, browser-based access to complex data and, therefore, the possibility to assist users in extracting subsets of relevant information for them. Recent examples include the Interactive Atlas^28^ of the Intergovernmental Panel on Climate Change (https://interactive-atlas.ipcc.ch, last access: 26 June 2024) and the Copernicus Interactive Climate Atlas created by the Copernicus Climate Change Service (https://atlas.climate.copernicus.eu/atlas, last access: 26 June 2024). The DeepMIP web application follows a similar approach by providing intuitive data access and custom workflows to simplify common model-data comparison tasks. The web application automatically calculates paleolocations for a single site or a list of present-day locations, extracts the corresponding model data from the various model grids and plots a summary of the results. The resulting data can be exported for further offline analysis, while the underlying Python code can be used as a starting point for custom analysis.

The dataset and tools provided are designed to enable data access for non-programmers and to streamline analysis for more advanced users to routinely evaluate existing and emerging paleoclimate data against the full DeepMIP-Eocene model ensemble. This will help to bridge the gap between modelling and data communities to ultimately advance our understanding of early Eocene climate and could potentially serve as a reference framework for similar projects of other geological time periods in the future.

## Methods

### DeepMIP-Eocene experiments

**Fig. 1:**
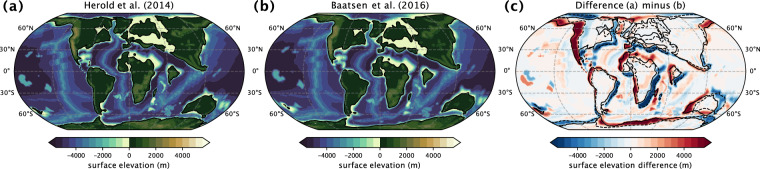
Comparison of available DeepMIP-Eocene paleogeographic boundary conditions. Orography and bathymetry are based on published reconstructions^29^ (**a**) and are also available based on a palaeomagnetic reference frame^30^ (**b**) with differences in the relative positions of plates (**c**).

All EECO simulations that follow the DeepMIP-Eocene experimental design protocol^12^ and were completed by September 2023 form the input data for version 1.0 of the dataset (Table 1). These simulations are identical to those described in the DeepMIP overview paper^13^, with the exception of the new MIROC ×1 and ×2 experiments. The DeepMIP framework provides standardised model boundary conditions and experimental designs to allow a coordinated model intercomparison of the simulation results. All groups have used one of the two reference paleogeographic reconstructions^29,30^ (Fig. 1a-b) interpolated to their respective model grids. The main difference between the two available paleogeographies is the choice of the applied rotation reference frame leading to slight differences in the relative positions of individual plates (Fig. 1c). Prescribed vegetation and river runoff follow a published reconstruction^29^, while globally homogeneous soil parameters based on the global mean of the respective pre-industrial simulation were used. All groups provided a pre-industrial reference simulation and performed a series of EECO experiments, differing only in the concentration of atmospheric CO_2_, summarised in Table 2. Other greenhouse gas concentrations and the solar constant were held constant at their pre-industrial levels.

**Table 2:** Overview of the DeepMIP-Eocene experiments included in version 1.0 of the dataset.

Experiment Name	Short Name	CO_2_ [ppmv]	Geography
deepmip-eocene-p1-PI	PI	280	modern
deepmip-eocene-p1-x1	x1	280	^29^ or^30^
deepmip-eocene-p1-x1.5	x1.5	420	^29^ or^30^
deepmip-eocene-p1-x2	x2	560	^29^ or^30^
deepmip-eocene-p1-x3	x3	840	^29^ or^30^
deepmip-eocene-p1-x4	x4	1120	^29^ or^30^
deepmip-eocene-p1-x6	x6	1680	^29^ or^30^
deepmip-eocene-p1-x9	x9	2520	^29^ or^30^

A complete overview of the modelling framework is given in the DeepMIP experimental design paper^12^, and detailed descriptions of its implementation in the individual models can be found in the analysis of the large-scale climatic features^13^. We also provide a full description of each model setup based on their published method sections^13^ as a README file in the dataset itself. This is intended to make the downloaded files self-describing and to allow dynamic addition of new experiments and models in the future. In the following, for each model included in version 1.0 of the dataset, we provide a brief summary of the initialisation and spin-up strategies, as this step required individual decisions by each modelling group. The DeepMIP experimental design provides an idealised equation for initialising the ocean temperatures as:

1 $$T{[}^{\circ }C]=\{\begin{array}{cc}\left(\frac{D-z}{D},\times ,A,\times ,\cos ,(,\,,\phi ,)\right)+B & {\rm{if}}\,{\rm{z}}\le {\rm{D}}\,{\rm{m}}\\ B & {\rm{if}}\,{\rm{z}} > {\rm{D}}\,{\rm{m}}\end{array}$$

where *ϕ* is latitude, and *z* is ocean depth. The parameters A, B and D are specified in the experimental design as 25, 15 and 5000, respectively^12^. The resulting warm ocean temperatures caused numerical problems in some model spin-ups and have therefore been modified for individual models. An overview of the parameters used for each model is given in Table 3. Any other deviations for the model initialisation are listed below.

**Table 3:** Overview of initial ocean temperature strategies.

Model	A	B	D	Comment
CESM1.2-CAM5	—	—	—	from previous CCSM3 simulation^31^
COSMOS-landveg-r2413	—	—	—	10 °C globally
GFDL-CM2.1	25	10	5000	
HadCM3B-M2.1aN	—	—	—	branched from HadCM3BL
HadCM3BL-M2.1aN	—	—	—	custom equations^13^
INM-CM4-8	20	15	5000	
IPSLCM5A2	25	10	1000	
MIROC4m	25	0	5000	
NorESM1-F	—	—	—	from previous NorESM-L simulation^34^
experimental design^12^	25	15	5000	

Coefficients A, B and D refer to Eq. (1).

#### CESM

Ocean temperatures and salinities in all Eocene simulations are initialised from the same Palaeocene-Eocene Thermal Maximum (PETM; ~55 million years ago) experiment using a previous version of CESM^31,32^. The ×1 simulation was integrated for a further 2600 years, while all other experiments were run for 2000 years. The mean top of the atmosphere (TOA) imbalance over the last 100 model years for the PI, ×1, ×3, ×6 and ×9 experiments are −0.05, −0.25, −0.32, 0.34 and 0.64 *Wm*^−2^, respectively.

#### COSMOS

The ×3 integration was initialised with a homogeneous temperature and salinity of 10°C and 34.7 psu, respectively, and integrated for an initial 1000 years, after which the ×1 and ×4 simulations were branched. After an initial 8000 years with transient orbital parameters, a constant, pre-industrial orbital configuration was used for the final 1500 years of all simulations. Instead of using the proposed river routing scheme^29^, the simulations use a hydrological discharge model that follows the model orography^33^. The mean TOA imbalance over the last 100 model years for the PI, ×1, ×3 and ×4 experiments are 1.75, 1.91, 1.78, and 1.95 *Wm*^−2^, respectively.

#### GFDL

The ×1, ×2, ×3, and ×4 simulations were started with a globally homogeneous salinity of 34.7 psu and a slightly cooler version of the DeepMIP temperature equation (Eq. (1); Table 3). After 1500 and 2000 years of integration, an acceleration technique was applied. Specifically, the linear temperature trends of the last 100 years for each model level below 500 m were calculated and the level-by-level temperatures were then extrapolated by 1000 years following this trend. After the second application of this technique at year 2000, the model was run out normally for a further 4000 years for a total of 6000 years. The ×6 simulation was initialised with a globally uniform temperature of 19.32^°^C and continously integrated for 6000 years. The mean TOA imbalance over the last 100 model years for the PI, ×1, ×2, ×3, ×4 and ×6 experiments are 0.31, 0.10, −0.08, −0.14, −0.19, and −0.28 *Wm*^−2^, respectively.

#### HadCM3

Initial ocean temperatures for HadCM3BL were derived from an idealised temperature profile with lowered, CO_2_ dependent deep ocean temperatures based on previous Eocene simulations. HadCM3B experiments were branched from the respective HadCM3BL simulations after 4400 to 4900 years and integrated for a further 2950 years. Multiple ocean gateways in the original paleogeography were widened to allow unrestricted ocean circulation and to guarantee the same gateway widths on both the low and high-resolution ocean grids of HadCM3BL and HadCM3B, respectively. In addition, maximum water depths in parts of the Arctic Ocean were reduced to improve numerical stability. The mean TOA imbalance per century averaged over the last 50 model years for the PI, ×1, ×2 and ×3 experiments for HadCM3B are −0.08, −0.02, −0.08 and −0.08 *Wm*^−2^, respectively.

#### INMCM

The ocean temperature and salinity in the ×6 simulation follow the idealised equations of the DeepMIP protocol, but with equatorial surface temperatures lowered by 5°C (Eq. (1); Table 3). The simulation was integrated for a total of 1150 years. The mean TOA imbalance over the last 100 model years for the PI and ×6 experiments are 4.37 and 2.87 *Wm*^−2^, respectively.

#### IPSL

A modified version of Eq. (1) with overall reduced subsurface temperatures (Table 3) and a globally homogeneous salinity of 34.7 psu were used to initialise the ×3 simulation. The ×1.5 simulation is branched from the ×3 experiment after 1500 years. Both simulations are run for a total of 4000 years. The ocean bathymetry around individual ocean straits has been manually adjusted to guarantee the minimum gateway width necessary to allow throughflow. The mean TOA imbalance over the last 100 model years for the PI, ×1.5 and ×3 experiments are 0.08, 0.59 and 0.76 *Wm*^−2^, respectively.

#### MIROC

All three simulations have been initialised with a modified version of the idealised DeepMIP temperature equation, with ocean temperatures globally reduced by 15°C (Eq. (1); Table 3), and integrated for 5000 model years. The ×1 and ×2 experiments are new and have not been included in the DeepMIP overview paper^13^. The mean TOA imbalance over the last 100 model years for the PI, ×1, ×2 and ×3 experiments are 0.96, 0.79, 0.91 and 0.96 *Wm*^−2^, respectively.

#### NorESM

Initial ocean temperatures for the ×2 simulation were used from a previous NorESM-L simulation^34^, while salinities were set to 25.5 psu in the Arctic and 34.5 elsewhere. The ×4 simulation was branched off after 100 model years, and both simulations have been run for a further 2000 years. The NorESM simulations were performed with a different paleogeographic reconstruction than the rest of the DeepMIP ensemble (Table 1). The mean TOA imbalance per century at the end of the PI, ×2 and ×4 experiments are −0.02, 0.03 and 0.24 *Wm*^−2^, respectively. Note that the PI imbalance is calculated over the last 1000 years, while the Eocene values are averaged over the last 100 years.

### Data processing

We use the raw output of the last 100 years of each of the 35 model simulations as input for our post-processing. For each variable, we generate up to three netCDF output files to facilitate common analysis workflows. We always produce a mean file representing either the monthly mean climatology or the annual mean averaged over the last 100 model years, depending on the temporal resolution of the model output. In case of monthly mean output data, the std file contains the standard deviation over the same averaging period for each month of the year and can be used for significance testing. Where feasible, we also store the full monthly mean output of the last 100 model years as a time_series file to investigate temporal trends or interannual variability.

Alongside this standard output, we provide a generic script to interpolate model fields from their native grids to a common resolution for model intercomparisons. The processing workflow requires a local installation of the Climate Data Operator (CDO) software^35^ for bilinear or nearest-neighbour interpolation for atmosphere and ocean variables, respectively. The processing script is distributed as part of the dataset (see Data Records section).

### Naming convention

We employ a consistent naming convention for variables, directories, and file names across all models to simplify the comparison of different models and to allow a scripted analysis of the entire dataset. The list of output variables is an extended version of those proposed in the DeepMIP experimental design^12^ and is shown in Tables 4-5. Variable names, units and signs of fluxes follow the naming convention of the Coupled Model Intercomparison Project 6 (CMIP6) data request (https://wcrp-cmip.github.io/WGCM_Infrastructure_Panel/CMIP6/data_request.html, last access: 26 June 2024). Consistent standard names, long names and global attributes are directly added to the netCDF files following the Climate and Forecast metadata conventions (CF^36^) in version 1.8 (http://cfconventions.org, last access: 26 June 2024). All netCDF file have been automatically tested for CF-compliance with the cf-checker utility (https://github.com/cedadev/cf-checker, last access: 26 June 2024) developed by the UK Met Office and the NCAS Computational Modelling Services (NCAS-CMS). Following the CMIP and CF community standards will both increase user familiarity with the new dataset and will allow the integration into existing analysis workflows and software. Each output variable is stored in a separate file according to the following structure:

**Table 4:** Atmosphere output variables included in version 1.0 of the dataset. Naming conventions follow the CMIP6 data request.

Name	Long Name	Units	Dimensions
tas	Near-Surface Air Temperature	*K*	time × lat × lon
ts	Surface Temperature	*K*	time × lat × lon
pr	Precipitation	*kgm*^−2^*s*^−1^	time × lat × lon
evspsbl	Evaporation Including Sublimation and Transpiration	*kgm*^−2^*s*^−1^	time × lat × lon
hfls	Surface Upward Latent Heat Flux	*Wm*^−2^	time × lat × lon
hfss	Surface Upward Sensible Heat Flux	*Wm*^−2^	time × lat × lon
ps	Surface Air Pressure	*Pa*	time × lat × lon
psl	Sea Level Pressure	*Pa*	time × lat × lon
snc	Snow Area Percentage	%	time × lat × lon
rsds	Surface Downwelling Shortwave Radiation	*Wm*^−2^	time × lat × lon
rlds	Surface Downwelling Longwave Radiation	*Wm*^−2^	time × lat × lon
rsus	Surface Upwelling Shortwave Radiation	*Wm*^−2^	time × lat × lon
rlus	Surface Upwelling Longwave Radiation	*Wm*^−2^	time × lat × lon
rsdt	TOA Incident Shortwave Radiation	*Wm*^−2^	time × lat × lon
rsut	TOA Outgoing Shortwave Radiation	*Wm*^−2^	time × lat × lon
rlut	TOA Outgoing Longwave Radiation	*Wm*^−2^	time × lat × lon
rsdscs	Surface Downwelling Clear-Sky Shortwave Radiation	*Wm*^−2^	time × lat × lon
rldscs	Surface Downwelling Clear-Sky Longwave Radiation	*Wm*^−2^	time × lat × lon
rsuscs	Surface Upwelling Clear-Sky Shortwave Radiation	*Wm*^−2^	time × lat × lon
rluscs	Surface Upwelling Clear-Sky Longwave Radiation	*Wm*^−2^	time × lat × lon
rsutcs	TOA Outgoing Clear-Sky Shortwave Radiation	*Wm*^−2^	time × lat × lon
rlutcs	TOA Outgoing Clear-Sky Longwave Radiation	*Wm*^−2^	time × lat × lon
tauu	Surface Downward Eastward Wind Stress	*Pa*	time × lat × lon
tauv	Surface Downward Northward Wind Stress	*Pa*	time × lat × lon
uas	Eastward Near-Surface Wind	*ms*^−1^	time × lat × lon
vas	Northward Near-Surface Wind	*ms*^−1^	time × lat × lon
clh	High Level Cloud Percentage	%	time × lat × lon
clm	Mid Level Cloud Percentage	%	time × lat × lon
cll	Low Level Cloud Percentage	%	time × lat × lon
clt	Total Cloud Cover Percentage	%	time × lat × lon
cl	Percentage Cloud Cover	%	level × time × lat × lon
hus	Specific Humidity	1	level × time × lat × lon
ta	Air Temperature	*K*	level × time × lat × lon
ua	Eastward Wind	*ms*^−1^	level × time × lat × lon
va	Northward Wind	*ms*^−1^	level × time × lat × lon
wap	Omega (=dp/dt)	*Pas*^−1^	level × time × lat × lon
zg	Geopotential Height	*m*	level × time × lat × lon
orog	Surface Altitude	*m*	lat × lon
sftlf	Percentage of the Grid Cell Occupied by Land	%	lat × lon


directory = deepmip-eocene-p1/<Family>/<Model>/<Experiment>/<Version>/<Averaging>/


filename = <Variable>_<Model>_<Experiment>_<Version>.<Statistic>.nc

- <Family>, <Model> and <Experiment> are listed in Tables 1 and 2, respectively
- <Variable> represents the first column in Tables 4-5

**Table 5:** Ocean output variables included in version 1.0 of the dataset. Naming conventions follow the CMIP6 data request.

Name	Long Name	Units	Dimensions
tos	Sea Surface Temperature	°*C*	time × lat × lon
siconc	Sea-Ice Area Percentage (Ocean Grid)	%	time × lat × lon
mlotst	Ocean Mixed Layer Thickness Defined by Sigma T	*m*	time × lat × lon
zos	Sea Surface Height Above Geoid	*m*	time × lat × lon
hfds	Downward Heat Flux at Sea Water Surface	*Wm*^−2^	time × lat × lon
wfo	Water Flux Into Sea Water	*kgm*^−2^*s*^−1^	time × lat × lon
tauuo	Sea Water Surface Downward X Stress	*Nm*^−2^	time × lat × lon
tauvo	Sea Water Surface Downward Y Stress	*Nm*^−2^	time × lat × lon
msftbarot	Ocean Barotropic Mass Streamfunction	*kgs*^−1^	time × lat × lon
msftmz	Ocean Meridional Overturning Mass Streamfunction	*kgs*^−1^	time × depth × lat
so	Sea Water Salinity	0.001	depth × time × lat × lon
thetao	Sea Water Potential Temperature	°*C*	depth × time × lat × lon
uo	Sea Water X Velocity	*ms*^−1^	depth × time × lat × lon
vo	Sea Water Y Velocity	*ms*^−1^	depth × time × lat × lon
wo	Sea Water Vertical Velocity	*ms*^−1^	depth × time × lat × lon
difvmo	Ocean Vertical Momentum Diffusivity	*m*^2^*s*^−1^	depth × time × lat × lon
difvtrbo	Ocean Vertical Tracer Diffusivity Due to Background	*m*^2^*s*^−1^	depth × time × lat × lon
deptho	Sea Floor Depth Below Geoid	*m*	lat × lon
- <Statistic> is either mean (1 or 12 timsteps), std (12 timsteps), time_series (1200 timsteps) or omitted for the time-independent boundary conditions
- the smaller mean and std files are stored in the <Averaging>=climatology directory and are separated from the larger time_series files in the <Averaging>=time_series directory to enable more granular download options

Storing all relevant information in the file name itself also allows new phases of coordinated DeepMIP simulations to be integrated into a single dataset in the future.

## Data Records

The full dataset has been deposited in the CEDA Archive, the UK national data centre for atmospheric and earth observation research^26^. This dataset contains the following types of files:

- **model data:** The directory deepmip-eocene-p1 contains all processed model output in CF compliant netCDF format^37^, a self-describing community standard for storing gridded simulation data, with a total file size of 168.0 GB. Directory and file structure follow the DeepMIP naming convention described above.
- **model READMEs:** Each <Family> top-level directory contains a single <Family>_README.md file that contains detailed information about the model, the simulation setup, and naming convention. This ensures the downloaded dataset is sufficiently self-described and allows the addition of new models and simulation results in the future.

In addition, the code of the web application^38^ and a collection of scripts and metadata to interact with the dataset^39^ are deposited in separate Zenodo repositories. The latter includes a collection of Python code to interpolate model data to a common grid (regrid_deepmip_data.py), recreate the validation tables of available data (plot_z-scores.py) and Python dictionaries containing available DeepMIP models, experiments and variables to support scripted analysis of the dataset.

## Technical Validation

**Fig. 2:**
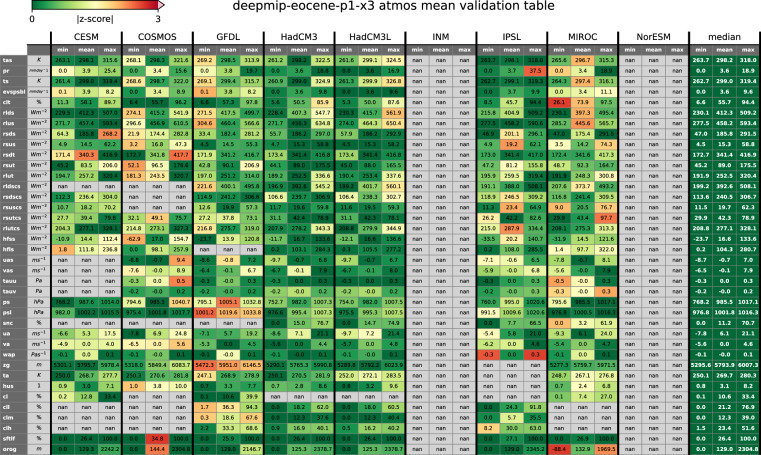
Technical validation of atmospheric global model fields of the ×3 experiment across the ensemble. Variables with multiple vertical levels are shown for the respective model pressure level closest to 500 hPa. Tables for other experiments and “time_series” files can be found in the web application at https://data.deepmip.org/Validation_tables. Note that the INM and NorESM models did not perform the ×3 experiment (Table 1) and are therefore excluded from this analysis.

**Fig. 3:**
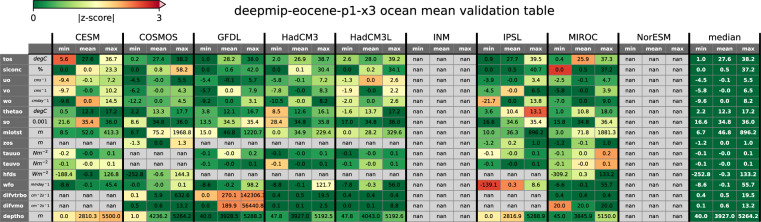
Technical validation of ocean global model fields of the ×3 experiment across the ensemble. Variables with multiple vertical levels are shown for the respective model depth closest to 1000 m. Tables for other experiments and “time_series” files can be found in the web application at https://data.deepmip.org/Validation_tables. Note that the INM and NorESM models did not perform the ×3 experiment (Table 1) and are therefore excluded from this analysis.

An earlier version of the dataset has already been used in a number of publications^13,15,16,18–21,24,25^ to assess the scientific validity of the model simulations, both in terms of model-model and model-data comparisons. In this section, we verify the internal consistency of the dataset, ensuring that the naming convention has been applied correctly and that the resulting variable names, units and fluxes are consistent across all models. To do this, we automatically parse all mean and time_series files in the dataset for any given experiment, interpolate them to a common grid, calculate the global mean, minimum and maximum values and compare these values across all models. We use annual mean fields for the validation of mean files and the last 12 available months of the time_series files. For variables with multiple vertical levels (see Tables 4-5), we select the vertical index nearest to the 500 hPa pressure level or 1000 m depth for atmospheric and ocean data, respectively. Example tables for atmospheric and ocean mean variables from the ×3 simulations are shown in Figs. 2 and 3, tables for all other experiments as well as for time_series files are uploaded to the web application. This testing procedure simulates a standard analysis workflow and is able to detect any deviations from the expected DeepMIP naming convention, while the resulting tables provide a visual overview of the available model fields for each experiment. We further calculate the median and standard deviation for each variable and metric across all available models (i.e. for each row in the table) to flag potential outliers that may arise due to inconsistent units or different directions of energy or mass fluxes. For this, we calculate a z-score for each model, variable and statistic which quantifies the number of standard deviations an individual model statistic is above or below the ensemble median. We use the ensemble median instead of the mean as the reference point to reduce the influence of potential outliers in our small sample sizes and calculate the adjusted z-scores as:

2 $$z=\frac{x-M}{\sigma }$$

where z is the computed z-score, *x* is the individual model value, M is the median across all available models for the respective variable and statistic (i.e., across each table row), and *σ* is the standard deviation across the ensemble. A z-score > 3 is commonly used as a cut-off to identify outliers in a distribution. Due to the small sample sizes (*N* ≤ 9) the z-score threshold was not used to exclude any data from the dataset, but rather to find and resolve inconsistencies in the data processing between the models. For this, the background of each cell in Figs. 2 and 3 has been coloured by their computed z-score to visually identify model results substantially different from the ensemble median. Note that all modelling groups have performed slightly different sets of simulations (Table 1) and not all models provide all requested output variables. These fields are indicated by gray “nan” cells in the overviw tables. For example, INM and NorESM did not perform a ×3 experiment and are therfore not included in Fig. 2 and Fig. 3. In the final dataset, all available model fields are within ±3 standard deviations around the respective ensemble median, although we note that the small sample sizes allow only an indicative analysis. The Python processing code is included in the online dataset (see Data Records section) and can be used to develop a custom analysis workflow or to validate any regridding and global averaging performed by the user.

## Usage Notes

We present two primary routes to access the dataset, either via downloading the netCDF files for local processing or via an interactive website for online model-data comparisons.

### netCDF repository

First, processed netCDF files for all simulations are available from the CEDA Archive^26^. The full directory structure can be accessed via the browser and files can be downloaded via HTTP, Wget, FTP or OPeNDAP. This allows easy access to the data via the browser, as well as scriptable interfaces for bulk downloading. The OPeNDAP (Open-source Project for a Network Data Access Protocol) protocol allows the remote subsetting and exploration of datasets directly in e.g. Python, R, IDL, and Matlab. The CEDA Archive website (https://help.ceda.ac.uk/article/99-download-data-from-ceda-archives; last access: 26 June 2024) provides an up-to-date overview of all available access options.

### Interactive web application

Second, simulated surface temperatures and precipitation from any location can be extracted, visualised and downloaded at https://data.deepmip.org. This allows model-data comparisons via a simple user interface without the need to download the netCDF files locally. The website is designed to extract surface temperature and precipitation for any user-defined location from all available model simulations and either visualise the results or download them for offline use. All processing code is written in Python and bundled into a web application via the Streamlit library (https://streamlit.io; last access: 26 June 2024). The code makes full use of the naming conventions described above and is therefore general enough to serve as a template for further in-depth analysis. The sidebar of the web application can be used to choose between three different analysis pages:

1. **Extract local model data:** Finds the model data closest to a user-specified site (see example in Fig. 4). The minimum inputs are the modern location of the site and the variable of interest (either near-surface air temperature, sea surface temperature, or total precipitation). The application will automatically reconstruct the site’s EECO paleo-position on both the mantle^29^ and paleomagnetic^30^ reference frames and extract the respective monthly and annual mean simulation data from the closest grid point for all models in the dataset. Model data is interpolated to a common 1°  × 1° grid (see Data processing section for details) prior to the data selection to eliminate the influence of different model resolutions on the results. In the end, the ensemble means for each experiment are calculated and the results are listed in an interactive table. Data can be downloaded in CSV, Excel or JSON format for direct import into spreadsheets for further offline analysis. The extraction can be performed for a single site or a list of locations and all sites from the DeepMIP proxy dataset^2^ are pre-loaded and available for comparison with the simulation results. Furthermore, the underlying Python functions get_paleo_locations() and get_model_point_data() are available in the deepmip_modules.py file of the application repository for reuse in any custom analysis. The get_paleo_locations() function uses the paleolocation lookup fields provided in the experimental design paper^12^ to find the respective early Eocene locations for a list of modern latitude/longitude pairs, using both the mantle^29^ and paleomagnetic^30^ reference frames. Results are saved in a Pandas DataFrame which can be directly passed to get_model_point_data() to extract the nearest model data for all reconstructed locations.

**Fig. 4:**
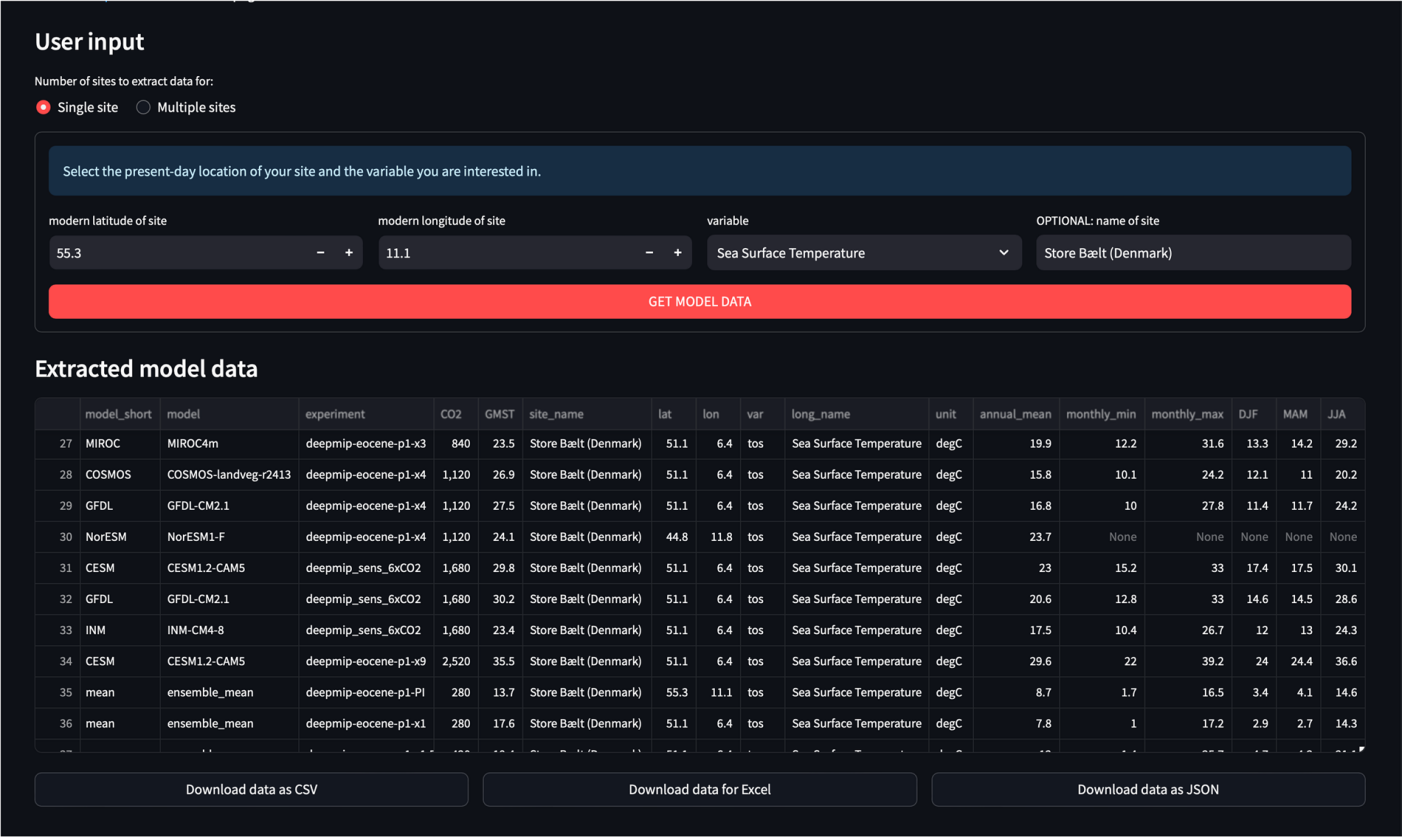
Example user input and extracted model data for a single site in the web application.
2. **Plot local model data:** Visualises the extracted results and optionally compares them to proxy reconstructions (see example in Fig. 5). Available visualisations include line plots of the annual cycle at the user-specified location, grouped by the various DeepMIP CO_2_ levels (Fig. 5a), and a scatter plot of all simulated annual mean values against the respective GMSTs or CO_2_ concentrations of the model simulations. (Fig. 5b). The latter plot type can be useful to compare the sensitivity of the model results at the local site against global climate signals. The simulated monthly and annual mean model results can be visually compared against a local proxy reconstruction, either by manually specifying the mean and standard deviation of the proxy data or by loading the respective values for locations from the DeepMIP proxy dataset^2^. The user can zoom and pan within the interactive figures and download them in PNG and SVG format.

**Fig. 5:**
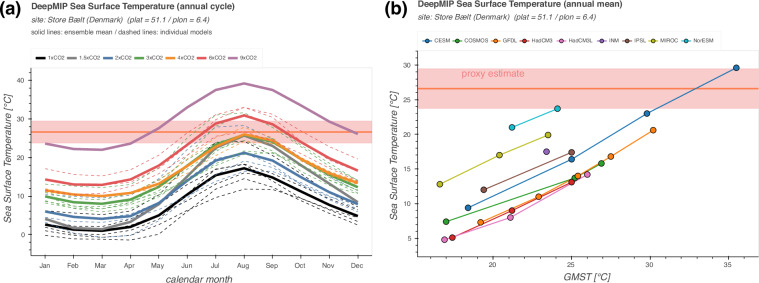
Example graphical output of the web application for the model-data comparison of the Store Bælt (Denmark) site defined in Fig. 4. (**a**) Simulated annual cycle of sea surface temperatures at the respective grid point closest to the reconstructed paleoposition of the site. Solid lines show the ensemble mean for each CO_2_ concentration with individual models represented by the dashed lines. (**b**) Scatter plot of the simulated annual mean sea surface temperature at the proxy site compared to the global mean surface temperature of the respective simulation. Lines connect results of the same model. Reconstructed proxy temperature is based on the TEX_86_ paleothermometer^2^.
3. **Map sites and boundary conditions:** Plots paleogeographic maps of the chosen site. The user can choose between a global map indicating the location of the study site or regional maps of the bathymetry, orography and land-sea mask on the various native model grids (Fig. 6). The latter can help with the interpretation of the model-data comparison result, e.g. by visualising local grid resolutions and associated intermodel differences in the representation of mountain ranges or ocean gateways.

**Fig. 6:**
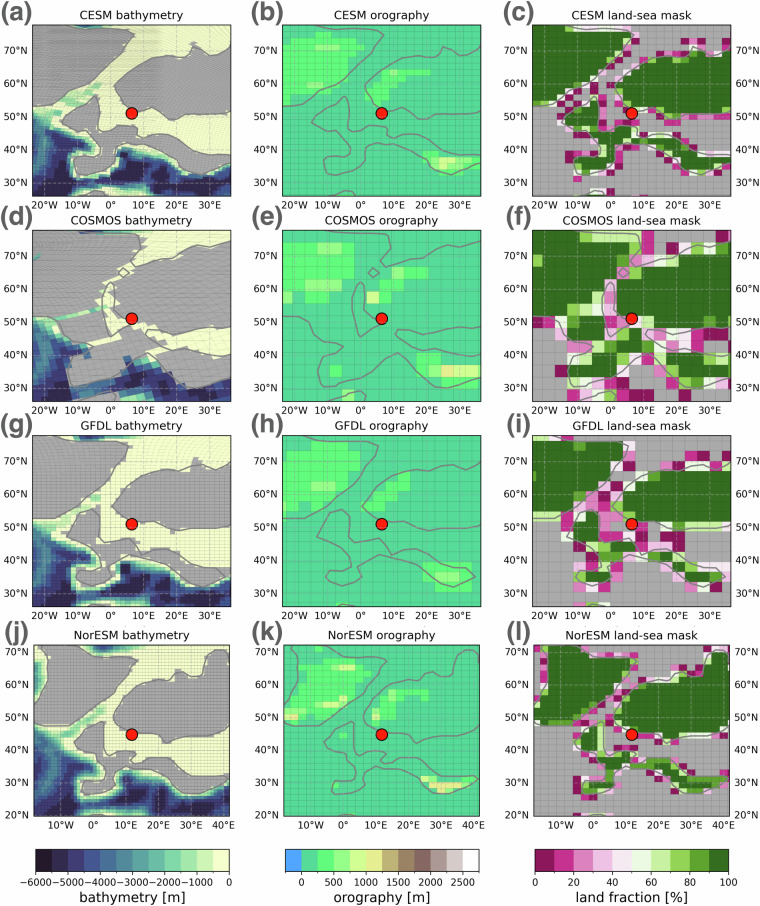
Maps of local boundary condition differences between some of the models around the the Store Bælt (Denmark) site defined in Fig. 4 produced by the web application. Note the different paleogeographic reconstruction used in NorESM (panel j-l).

### How to cite the dataset

This Data Descriptor paper should be cited whenever any netCDF files from the dataset or results from the web application are reused in a publication. In addition, the user might want to cite the previously published overview of simulated large-scale climate features^13^ or the DeepMIP-Eocene experimental design^12^, as appropriate.

## Data Availability

Processing code to interpolate model fields and to create the validation overview tables is available via Zenodo^39^. The code for the web application is deposited in a separate Zenodo repository^38^.
